# Recurrence of solitary fibrous tumor of the pleura with hypoglycemia (Doege–Potter Syndrome): a case report description

**DOI:** 10.3389/fonc.2023.1245289

**Published:** 2023-09-26

**Authors:** Chuxu Wang, Tingting Cao, Guodong Hu, Bo Min, Haibo Hu, Bing Liu, Yaqin Wang, Xiaohua Zuo

**Affiliations:** ^1^ Department of Thoracic Surgery, Huai’an Second People’s Hospital, The Affiliated Huai’an Hospital of Xuzhou Medical University, Huai’an, Jiangsu, China; ^2^ Department of Pain Management, Huai’an Second People’s Hospital, The Affiliated Huai’an Hospital of Xuzhou Medical University, Huai’an, Jiangsu, China

**Keywords:** solitary fibrous tumors, pleura, Doege-Potter syndrome, hypoglycemia, paraneoplastic syndrome

## Abstract

Hypoglycemia has multiple causes, but the most common is a complication of insulin treatment. In addition to insulin therapy, tumors such as insulinomas of pancreatic origin and extrapancreatic tumors causing paraneoplastic syndromes should also be considered. Solitary fibrous tumors of the pleura (SFTP) is rare tumor, which when associated with hypoglycemia causes Doege-Potter syndrome. This article reports a case of a 69-year-old man with Doege-Potter syndrome and underwent the first surgical resection for SFTP. However, the tumor recurred 9 years later with hypoglycemic symptoms and implant metastasis. This recurrent tumor originated from the visceral pleura, was more aggressive and invaded the diaphragm and parietal pleura. After the second surgical removal of the tumor, the hypoglycemic symptoms disappeared.

## Introduction

Hypoglycemia can be caused by several factors, including insulin treatment for diabetes, congenital hyperinsulinism, insulinomas and extrapancreatic tumor (1). Among them, extrapancreatic tumor accounts for approximately 25% of all hypoglycemic cases (2). Hypoglycemia is characterized by central nervous system symptoms, such as confusion, headaches and palpitations (3). Therefore, hypoglycemia caused by tumors is not easily detected. Solitary fibrous tumor of the pleura (SFTP) is a rare type of tumor, accounting for approximately 5% of all pleural tumors (4). SFTP is most commonly localized in the pleura but can arise anywhere in the body (5). The tumor also can produce symptoms such as hemorrhage, digital clubbing, hypertrophic pulmonary osteoarthropathy and hypoglycemia (4). When hypoglycemia is associated with SFTP, it is known as the Doege-Potter syndrome (6). This rare paraneoplastic syndrome is often diagnosed incidentally during the evaluation of hypoglycemia with unclear etiology (7).

## Case description

**Figure 1:**
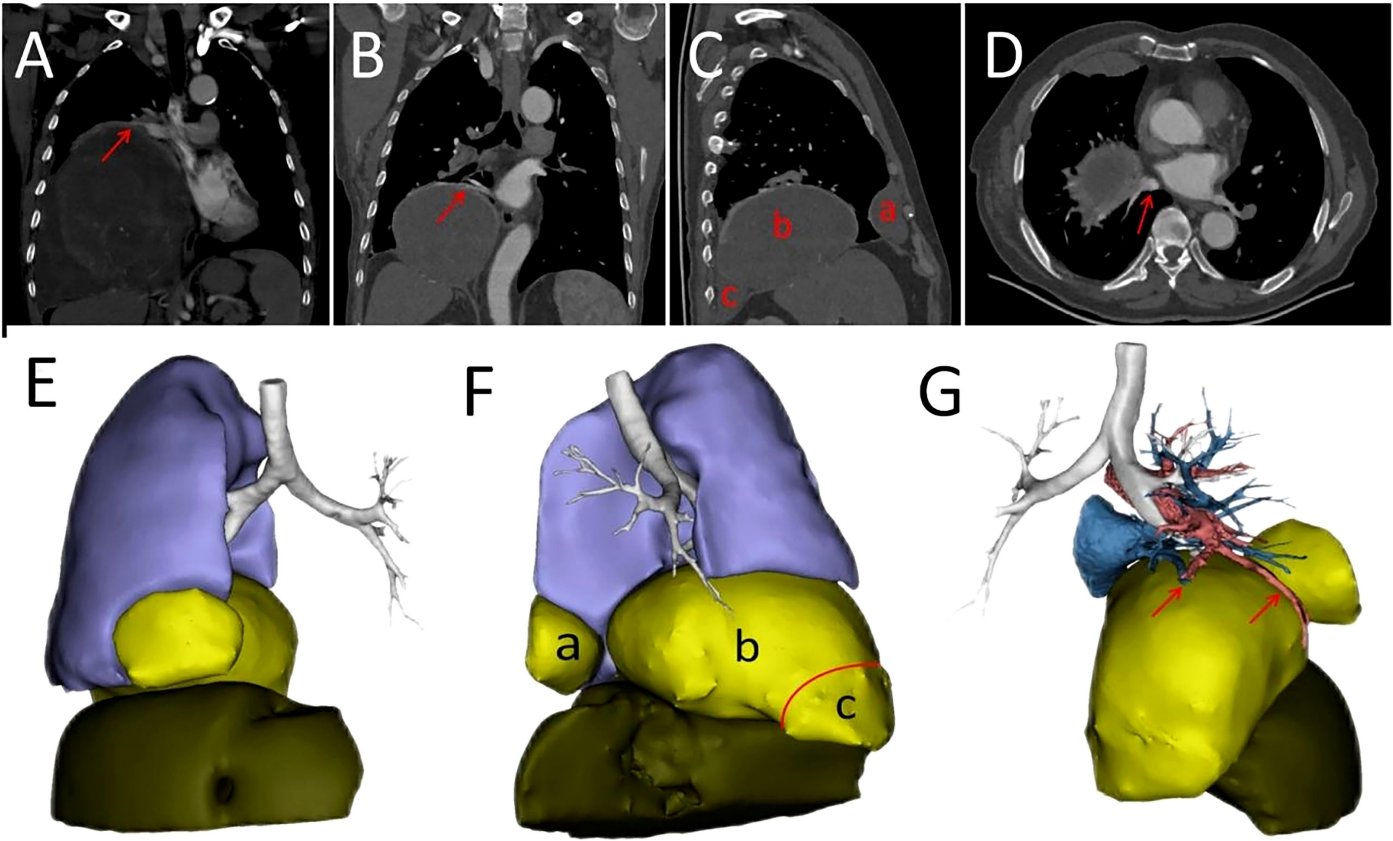
**(A)** Contrast-enhanced CT scan showing tumor before first surgery of the patient. The arrow represents a rim enhancement, i.e., tumor-feeding vessels around the tumor. **(B)** Tumor-feeding vessels originated from the inferior pulmonary vein shown by an arrow. **(C)** The tumor developed recurrence (b) and implant metastases on anterior (a) and posterior (c) pleura. **(D)** The tumor adjacent to the inferior pulmonary vein as indicated by an arrow. The tumor adjacent to the right lower lobe, right middle lobe of the lung and the diaphragm. **(E)** Anterior view of a three dimensional (3D) reconstruction. **(F)** Left view of a 3D reconstruction. Not tight junctions are indicated by the red arc. The tumor originated from posterior pleura (red triangle). Recurrent tumor (b) and implant metastatic tumors (a, c) **(G)** The relationship between the tumor and the vessels. The blood vessels supplying the recurrent tumor are indicated by arrows.

Our patient was a 69-year-old male who presented to our hospital with symptoms of confusion. He had no significant past medical history or family history of chronic disease. During his evaluation, electroencephalogram (EEG) examination initially precluded epilepsy syndrome. His blood glucose was 2.8 mmol/L (normal: >3.9mmol/L), which was relieved with emergency injection of 40 ml of 50% dextrose. Upon further questioning, we found that the patient had similar symptoms 9 years ago. At the time, he was admitted to the hospital for confusion and found to be hypoglycemic, but diabetes and insulinomas were precluded. A huge tumor in his right chest cavity was found during a computed tomography (CT) scan. Analysis of the past diagnostic imaging revealed a 20 cm × 18 cm mass lesion in the right hemithorax (**Figure 1A**). After the whole tumor was surgically removed, his symptoms never recurred. Postoperative pathologic examination confirmed the final diagnosis of SFTP. At 2-year follow-up after the surgery, no tumor recurrence was found on chest CT. However, the patient was subsequently lost to follow-up until he presented with re-occurrence of symptoms of confusion. During this hospitalization, a mass tumor in the right hemithorax was found with the contrast-enhanced chest CT (**Figures 1B–D**). On diagnostic imaging, the tumor was found to originate from the visceral pleura of the right lower lung with the rim-enhancement (**Figure 1B**) and had implantation metastasis spread to the parietal pleura (**Figure 1C**). The imaging results guided the separation between the tumor and inferior pulmonary vein to avoid vessel injury (**Figure 1D**).

**Figure 2:**
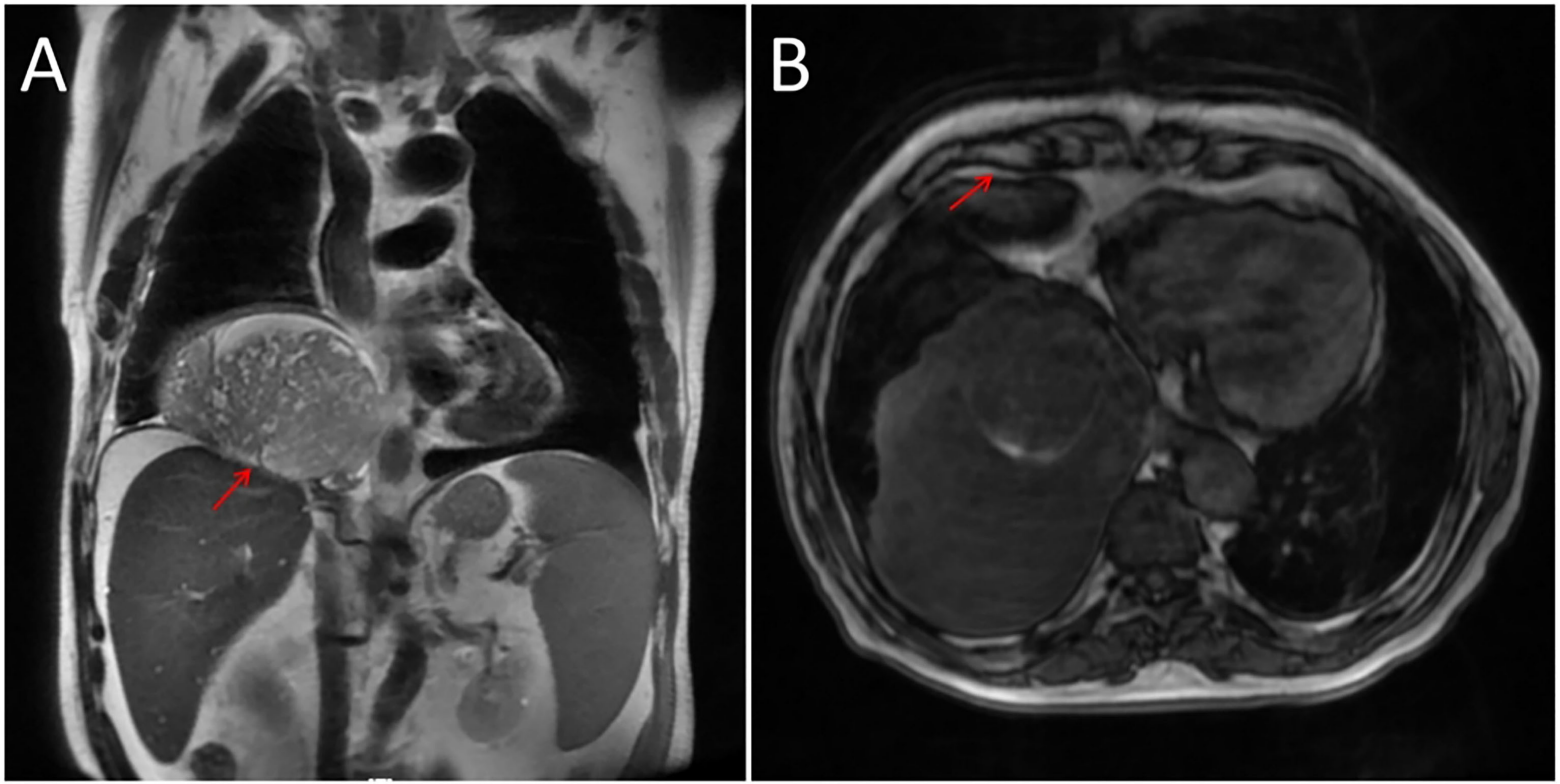
**(A)** Adhesion between the tumor and diaphragm as indicated by an arrow. **(B)** Arrow represents loose junctions between the tumor and anterior pleura.

**Figure 3:**
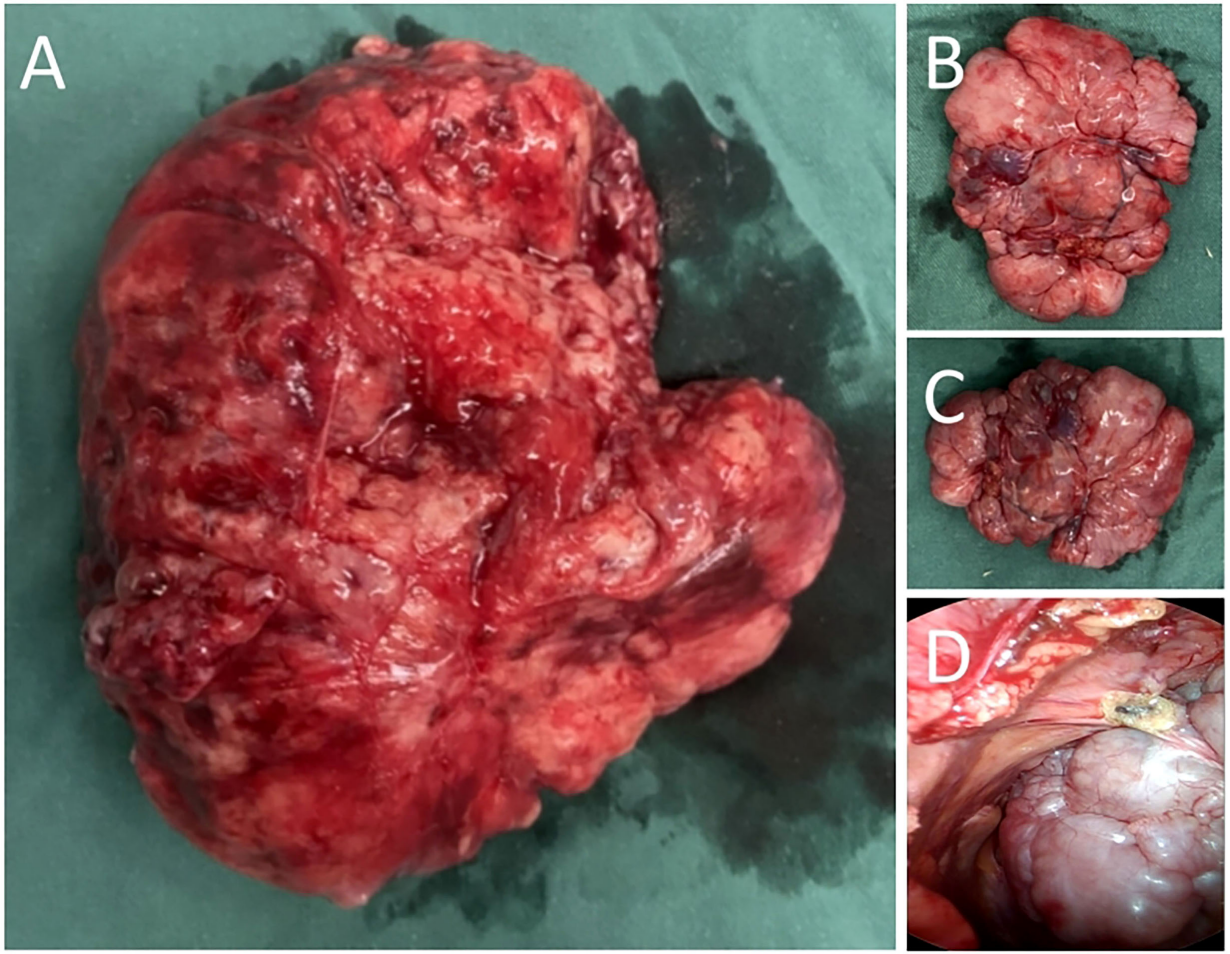
**(A)** The tumor arising from right lower lung. **(B)** Seeding tumors of anterior pleura. **(C)** Seeding tumors of posterior pleura. **(D)** Thoracoscopic view of the tumor located in the posterior pleura.

**Figure 4:**
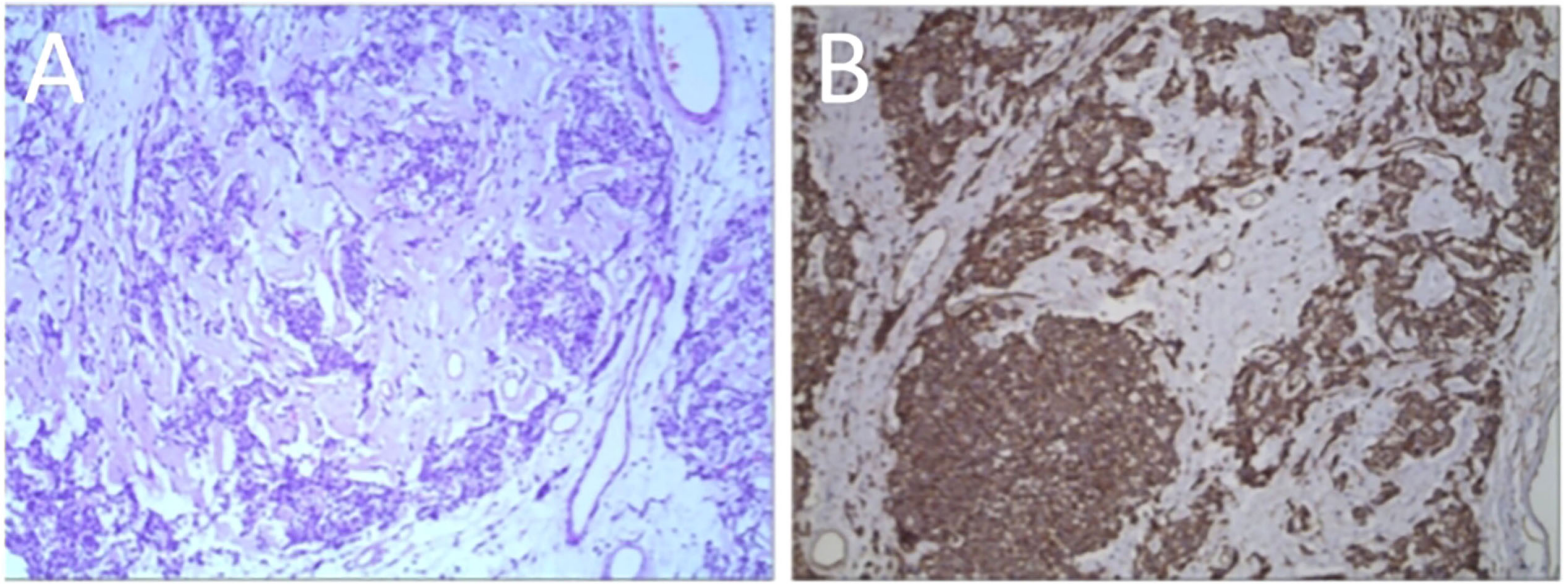
**(A)** Tumor cells and collagen. **(B)** Positive immunohistochemistry staining for CD34.

In addition, a three-dimensional (3D) reconstruction was performed to understand the relationship between the tumor and its surrounding tissues, including blood vessels, lung and liver (**Figures 1E–G**). A 3D reconstruction of the relationship among the three tumors is shown in **Figure 1F**. The blood supply of larger tumors is mainly from a pulmonary artery and vein (**Figure 1G**). Magnetic resonance imaging (MRI) showed that the tumor had invaded the diaphragm and was not closely connected with parietal pleura (**Figure 2**). We performed IGF-1 and IGF-2 assays on the patient’s serum from the day before surgery. Serum levels of IGF-1 and IGF-2 were measured at 53.20 ng/mL (normal: 69-200 ng/mL) and 732.0 ng/mL (normal: 396.0-1,049.0 ng/mL), respectively and the ratio of IGF-2/IGF-1 is 13.76. Other markers were not found to be suppressed, including serum C−peptide 1.72 ng/ml (normal: 1.1-4.4 ng/mL), insulin 39.30pmol/L (normal: 17.8-173 pmol/L). After being informed about associated risks, the patient consented to repeat surgery. During the operation, the blood vessels between the right lower lung and tumor were visible and the tumor was closely adherent to the diaphragm. To ensure a complete resection of the tumor, part of the diaphragm and parietal pleura adhesions to the tumor was removed, revealing that the tumor had not invaded the abdominal liver. The diaphragm was sutured intermittently because the defect was not large. Ultimately, we found that the tumor consisted of three parts, which adhered to the right lower lung, anterior and posterior pleura (**Figures 3A–C**). The implanted tumor mainly received blood supply from the blood vessels in the chest wall (**Figure 3D**). The patient had an uneventful postoperative recovery and was extubated and discharged from the hospital on postoperative day (POD) 4 and POD 7, respectively. Postoperative pathology indicating SFTP (**Figure 4**). CD34, Bcl-2 and CD99 expression were positive by immunohistochemistry and expression > 15% by Ki–67. The mitotic count was higher than two mitoses per 10 high-power fields.

## Discussion

Solitary fibrous tumors are rare mesenchymal neoplasm tumors that usually arise in the pleura. About 4% of these tumors are associated with Doege–Potter syndrome (8). In the present patient case, after the first surgery, the tumor recurred in the visceral pleural and spread to the parietal pleura as implant metastases.

Typically, SFTP are occasionally detected on imaging examinations, as they have no significant clinical symptoms. However, when the tumor is large and compresses adjacent lung lobes or invade pleural, it can produce symptoms such as coughing, dyspnea and chest pain (9). In rare cases, the tumor can also produce symptoms such as hemorrhage, digital clubbing, hypertrophic pulmonary osteoarthropathy and hypoglycemia. When SFTP secretes excessive insulin-like growth factor II (IGF-2), it can cause a rare recurrent hypoglycemia called Doege–Potter syndrome (10). These symptoms usually gradually disappear after tumor resection, but they could reappear when tumor recurs (11). Although these symptoms reappeared in our patient case, the expression of IGF-2 was not significantly high. The main reason for this is that IGF-2 levels frequently fall within the normal range in non-islet cell tumor hypoglycemia (NICTH). Therefore, the IGF-2/IGF-1 ratio serves as a surrogate marker for the concentration of large IGF-2. A ratio of >10 is considered clinically significant (12, 13). Prepro IGF-2 is cleared to form IGF-2 material and Prepro IGF-2 molecules (known as “big IGF-2”) have also been shown to have biological activity and to be capable of producing hypoglycaemia in the setting of a normal IGF-2 level (12, 14). With regard to the diagnostic method for IGF-2, we mainly use serum. According to literature, both serum and plasma from patients were sampled for measurement (12, 13).

SFTP is diagnosed mainly using contrast-enhanced chest CT and MRI. Small SFTP usually has no specific representation on imaging but is characterized by soft-tissue, non-invasive masses in the pleura (4). When SFTP enlarges, it typically compresses surrounding tissues and sometime presents with low attenuation and calcification in the central area because of necrosis, hemorrhage and myxoid change (9). On MRI, SFTP usually demonstrates low or intermediate intensity T1 and T2 signals but can sometimes show as high signal intensity on T2-weighted images. Therefore, MRI can be useful for assessing the relationship between a tumor and its surrounding tissue, including chest wall and diaphragm (4).

However, the definitive diagnosis and differential diagnosis of SFTP primarily rely on pathology and immunohistochemistry. Specifically, the SFTP test is positive for vimentin, CD 34 and/or BCL-2, and it is nonreactive for desmin and S-100 (5). The malignancy diagnosis is confirmed by histologic criteria, including high mitotic activity, increased pleomorphism, necrosis, and associated pleural effusion (9). In a previous large-scale study, England et al. have defined the criteria of malignancy, which includes elevated mitotic activity of higher than 4 mitotic figures per 10 high-power fields (9). In the present case, the tumor recurred after the first surgery with evidence of implantation metastasis in the diaphragm and parietal pleura. The information analysis above suggests that the tumor is not malignant but may still be recurrence or metastasis, which is consistent with other study (15). It is known from the literature that immunohistochemistry is mainly used for diagnosis and differential diagnosis, and has little significance for treatment (9). Indeed, we have found in other study that IGF-1R (insulin-like growth factor-1 receptor) and its ligands, mainly IGF-1 and IGF-2, have been proposed to play key roles in tumor cell proliferation, survival and migration in other tumors such as colon cancer. Moreover, the cyclolignan picropodophyllin (PPP) has a good inhibitory effect on IGF-1R (15). If IGF-1R or its associated factors is expressed in SFTP, this may provide new sight for its target therapy.

At present, complete surgical resection is the main treatment for SFTP. The primary aim of surgery is to achieve radical surgical excision of the tumor with 1-2 cm margins because of its recurrent characteristics (9). For recurrent tumor, repeat resection is recommended (9). Our patient’s resection margin was negative because part of the diaphragm and parietal pleura adhesions to the tumor was removed. But its margin may not be enough to 1-2 cm. Radiotherapy and chemotherapy are not optimal treatments due to the low cellular content and low mitotic rates of SFTP. Adjuvant therapy is still recommended for tumors that margins was close or positive (9). A present study found that temozolomide plus bevacizumab is a safer and effective treatment for long-term management of malignant SFTP (16, 17). In addition, brachytherapy and photodynamic therapy, which is used to treat malignant mesothelioma, can also be used to treat SFTP which cannot be completely removed (18). Follow-up chest CT are recommended for all patients postoperatively, every 6 months for 2 years, and yearly thereafter (9). Unresectable tumors presenting with hypoglycemia should require regular glucose and IGF testing. Overall, patients with SFTPs have a better prognosis because the tumors mostly present with benign behavior. Nevertheless, a some patients still die due to extensive growth and recurrence (9). Therefore, early diagnosis and treatment of SFTP and close postoperative follow-up are important for a better prognostic outcome.

## Data availability statement

The original contributions presented in the study are included in the article/supplementary material. Further inquiries can be directed to the corresponding authors.

## Ethics statement

The studies involving human participants were reviewed and approved by the ethics committee of the Affiliated Huai’an Hospital of Xuzhou Medical University. The studies were conducted in accordance with the local legislation and institutional requirements. The patients/participants provided their written informed consent to participate in this study. Written informed consent was obtained from the individual(s) for the publication of any potentially identifiable images or data included in this article.

## Author contributions

All authors made a significant contribution to this report. CW and TC drafted the manuscript. YW and XZ revised the manuscript. All authors contributed to the article and approved the submitted version.
